# RecG controls DNA amplification at double‐strand breaks and arrested replication forks

**DOI:** 10.1002/1873-3468.12583

**Published:** 2017-02-28

**Authors:** Benura Azeroglu, David R. F. Leach

**Affiliations:** ^1^ Institute of Cell BiologySchool of Biological SciencesUniversity of EdinburghUK

**Keywords:** DNA amplification, double‐strand break repair, RecG

## Abstract

DNA amplification is a powerful mutational mechanism that is a hallmark of cancer and drug resistance. It is therefore important to understand the fundamental pathways that cells employ to avoid over‐replicating sections of their genomes. Recent studies demonstrate that, in the absence of RecG, DNA amplification is observed at sites of DNA double‐strand break repair (DSBR) and of DNA replication arrest that are processed to generate double‐strand ends. RecG also plays a role in stabilising joint molecules formed during DSBR. We propose that RecG prevents a previously unrecognised mechanism of DNA amplification that we call reverse‐restart, which generates DNA double‐strand ends from incorrect loading of the replicative helicase at D‐loops formed by recombination, and at arrested replication forks.

## Abbreviations

**cSDR**, constitutive stable DNA replication

**DSBR**, double‐strand break repair

**DSBs**, DNA double‐strand breaks

**iSDR**, inducible stable DNA replication

**SIOD**, Schimke immunoosseous dysplasia

Over the years since its discovery, different hypotheses have been put forward to explain the function of RecG in bacteria. These have ranged from branch migration and resolution of Holliday junctions 1, 2, 3, 4, 5, 6 via the promotion and inhibition of RecA‐mediated strand exchange 7, 8 to replication fork reversal 9, 10, 11, 12, 13, 14, 15. However, evidence has recently emerged that RecG is implicated in stabilising joint molecules 16 and in controlling DNA amplification by a mechanism that involves over‐replication associated with DNA double‐strand break repair (DSBR) 17, 18, 19, 20, 21, 22, 23, 24. These observations place RecG at the interface of DNA replication and DNA repair. But what is the function of RecG? Four hypotheses have been proposed to account for the role of RecG in preventing over‐replication. In two of these, RecG prevents the formation of DNA double‐strand ends that are associated with the generation of new origin‐independent replication forks by two different mechanisms 17, 21. In the third hypothesis, RecG catalyses the formation of double‐strand ends that are associated with the elimination of new origin‐independent replication forks 23. And in the fourth hypothesis, RecG prevents a form of origin‐independent DNA replication known as constitutive stable DNA replication (cSDR), which is initiated at R‐loops 25.

For many years, no eukaryotic homologue or orthologue of the bacterial RecG protein had been identified. However, recently several candidates have been proposed. These include the mitochondrial helicase Irc3 of *Saccharomyces cerevisiae* 26, the plastid and mitochondrial helicase RECG of *Physcomitrella patens* 27, the mitochondrial helicase RECG1 of *Arabidopsis thaliana* 28 and the human nuclear helicase SMARCAL1 29. All of these genes are implicated in the maintenance of DNA stability and all the plastid and mitochondrial genes show partial cross‐complementation with *recG*. Irc3 and SMARCAL1 catalyse similar reactions to purified RecG on replication fork and Holliday junction substrates *in vitro*. SMARCAL1 is a particularly attractive orthologue of RecG as it is a nuclear DNA damage response protein that is a substrate for phosphorylation by ATR 30, 31 and travels with the replication fork 32. Cells lacking SMARCAL1 are prone to accumulate DSBs 32 and patients with a biallelic deficiency in *SMARCAL1* have the Schimke immunoosseous dysplasia (SIOD) disease that includes cancer predisposition 33, 34. It is interesting to note that SMARCAL1 is required to accurately and effectively replicate telomeric DNA 35, 36, 37. This is the DNA of eukaryotic chromosomes that is predicted to be most sensitive to replication restart because a stalled replication fork at this location cannot be rescued by a convergent fork from another replication origin.

In this review, we firstly discuss the importance of DNA double‐strand breaks (DSBs) in DNA amplification. We then describe the evidence that RecG and RuvABC catalyse alternative steps in DNA repair by homologous recombination. This is followed by an overview of the biochemical activity of RecG and a discussion of whether the replication fork reversal reaction, which has been well documented to be catalysed by RecG *in vitro*, is implicated in DNA repair *in vivo*. We then discuss the recent evidence that RecG and RuvABC collaborate to stabilise joint molecules. Finally, we discuss the evidence that RecG prevents DNA amplification at DSBs and arrested DNA replication forks and assess the strengths of the four models that have been proposed to account for the function of RecG. Readers are encouraged to consult two recent reviews that take different perspectives. In the first of these, Piero Bianco concentrates on the biochemical activities of the protein with a particular emphasis on recent single‐molecule approaches to studying replication fork reversal catalysed by RecG 38. In the second, Christian Rudolph and colleagues discuss chromosome replication in the absence of RecG concentrating on the hypothesis that replication fork collisions are responsible for ‘pathological’ patterns of DNA replication and on the role of replication fork traps (where the Tus protein binds *ter* sites) in this context 39.

In eukaryotic cells, DSBs associated with DNA replication stimulate DNA amplification highlighting the importance of understanding the sources of replication‐dependent DSBs and their association with over‐replication.

**Figure 1:**
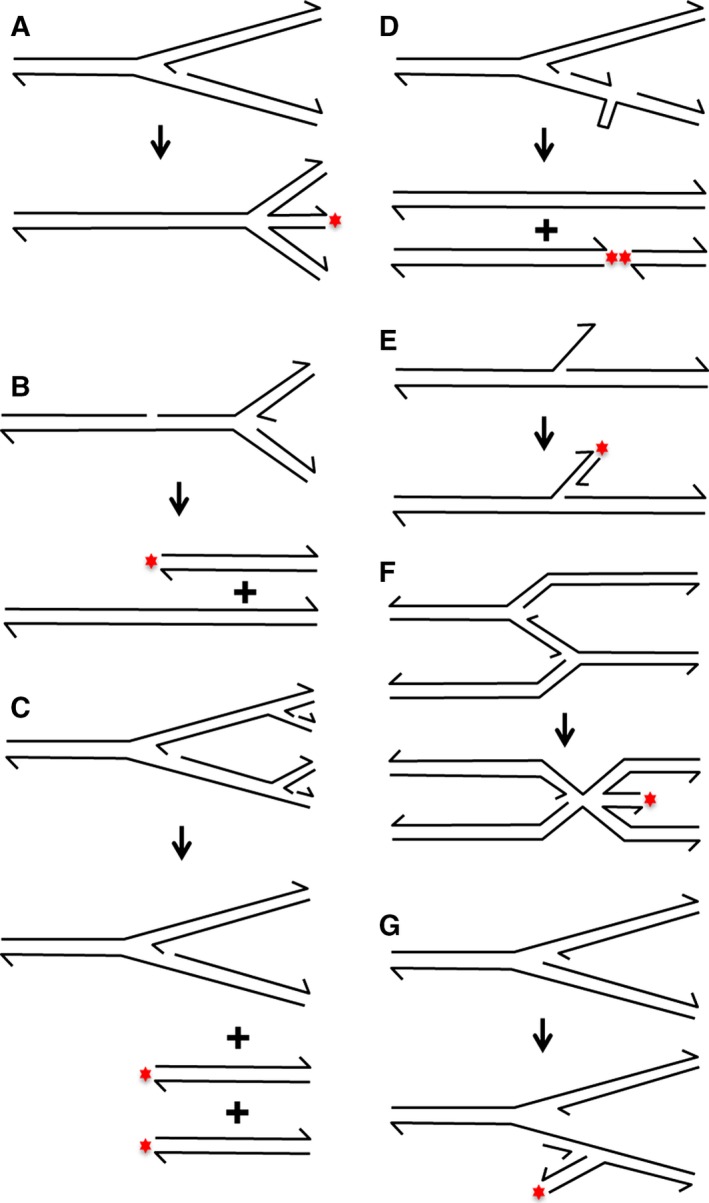
Sources of DNA double‐strand breaks formed during DNA replication. Red stars indicate the positions of DNA double‐strand ends. (A) Replication fork reversal. A four‐way ‘chicken‐foot’ structure can be generated when parental DNA strands re‐pair and newly replicated strands anneal. This forms a DNA double‐strand end and a Holliday junction, which may be cleaved to generate a broken chromosome 55, 56, 57. (B) Replication fork collapse. A one‐ended DSB can be generated when a DNA replication fork encounters a nick on one of the template strands 58. (C) Replication fork rear‐ending. Two one‐ended DSBs can be formed when a DNA replication fork is arrested and the subsequent DNA replication forks replicate this arrested fork 59. (D) Secondary structure cleavage. A DNA secondary structure, such as a hairpin, may form during DNA replication. A two‐ended DSB can be generated when a structure‐specific nuclease, such as SbcCD (Rad50/Mre11), cleaves this sequence 60. (E) Replication fork restart at a 3′ flap. A one‐ended DSB may be formed if a 3′ flap is generated during the termination of DNA replication and acts as a template for initiation of DNA synthesis and the assembly of a new replication fork 21. (F) Template‐switching with replication fork reversal. Template‐switching may occur when two replication forks collide. The two newly replicated strands would then act as reciprocal templates, which would result in DNA over‐replication. To eliminate this over‐replication, one of the replication forks might reverse, forming a DNA double‐strand end that can be degraded 23. (G) Reverse‐restart of an arrested replication fork. Following replication fork arrest, incorrect loading of the replicative helicase to a newly replicated DNA strand would result in the establishment of a new fork proceeding in the reverse direction. This reaction would generate a DNA double‐strand end 17.

DNA amplification, the formation of an abnormally high copy number of one or more genomic regions, is a characteristic of cancer and of the evolution of tumours that resist treatment with anticancer drugs 40, 41, 42, 43. It is also a mechanism that bacteria use to evolve resistance to antibiotics 44. There is evidence that in eukaryotes DNA amplification is stimulated by impaired S‐phase checkpoint activities and by chromosomal sites and treatments that elevate the frequency of DNA double‐strand ends associated with DNA replication 45, 46, 47, 48, 49, 50. These amplification events are frequently associated with altered deoxynucleoside triphosphate pools and DNA replication stress leading to the early stages of cancer development 50, 51, 52, 53, 54. For these reasons, it is critical to understand the pathways by which DNA double‐strand ends are formed as a consequence of DNA replication and how these events may be associated with DNA amplification. Many of these pathways of DNA double‐strand end formation have been initially investigated in prokaryotic systems but are not exclusive to prokaryotes. As depicted in Fig. 1, the pathways of replication‐dependent DSB formation include: (A) replication fork reversal 55, 56, 57, (B) replication fork collapse 58, (C) replication fork rear‐ending 59, (D) secondary structure cleavage 60, 61, (E) replication fork restart at a 3′ flap 21, (F) template‐switching with replication fork reversal 23, and (G) reverse‐restart of an arrested replication fork 17. Depending of the pathway, RecG has been proposed to promote the formation of double‐strand ends (in pathways A and F) or to prevent the formation of double‐strand ends (in pathways E and G). Pathways E and G postulate over‐replication associated with the formation of DSBs invoking a direct link between DSBs and DNA amplification in *Escherichia coli*. We will evaluate below the arguments for and against the proposed *in vivo* roles of RecG.

## RecG and RuvABC catalyse alternative steps in DNA repair and recombination

The *recG* gene was first identified by Storm and collaborators as a recombination‐deficient mutant of *E. coli* K12 62. Cells with the *recG162* or *recG258* mutation were more sensitive to UV, ionising radiation and mitomycin C, and displayed reduced conjugational and P1 transductional efficiency 1, 62, 63. More recent *in vivo* studies have confirmed the involvement of RecG in DSBR. Cells lacking RecG are sensitive to breaks induced by the I‐SceI homing endonuclease 5, the *Eco*KI endonuclease 6 and cleavage of a 246 bp palindrome by the SbcCD DNA hairpin endonuclease 60. The observation that (like RecA) RecG plays a role in several different homologous recombination pathways in *E. coli* suggests that it plays a fundamental role in DNA repair 63. But, what does RecG do? Further understanding of the role of RecG came from genetic studies combining the *recG* mutation with other mutations in genes encoding proteins involved in DNA repair and recombination 1, 63. *recG* mutants showed a modest additional sensitivity to UV when combined with either the *recB* (RecB subunit of the RecBCD enzyme, exonuclease IV, implicated in DNA double‐strand end unwinding, resection and RecA loading during DSBR) or *recJ* (RecJ 5′–3′ exonuclease, implicated in gap extension during single‐strand gap repair) but not the *recF* mutation (RecF component of RecFOR, implicated in RecA loading during single‐strand gap repair). However, more striking observations were obtained when *recG* was combined with *ruv* mutations (RuvABC implicated in the branch migration and cleavage of Holliday junctions). Double *ruvA recG, ruvB recG* and *ruvC recG* mutants exhibited a more dramatic increase in sensitivity to UV and ionising radiation, and a greater defect in recombination after conjugation or transduction when compared to either of the single mutants. These results suggest that RecG and RuvABC catalyse two alternative steps in the repair of DSBs by homologous recombination, potentially during the resolution of Holliday junctions 1. This idea was supported by the study of *rusA* mutants that suppress the recombination deficiency phenotype of *ruvA* mutants. These suppressor strains have activated the expression of a Holliday junction resolvase gene encoded within a cryptic prophage 4. The suppression observed in these *ruvA rusA* double mutants requires the presence of RecG, further suggesting that the alternative pathways catalysed by RuvABC or RecG might be for the resolution of Holliday junctions 4. However, we describe below an alternative hypothesis to explain the redundancy of RecG and RuvABC.

## RecG protein unwinds and remodels branched DNA molecules *in vitro*

Purified RecG protein has 3′–5′ helicase and nucleic acid translocase activities. *In vitro*, it can bind and unwind synthetic model Holliday junctions and various other types of branched DNA substrates including replication forks, D‐loops and R‐loops 2, 3, 8, 9, 10, 64, 65, 66, 67, 68. Unlike most other helicases, this enzyme unwinds DNA by translocating on dsDNA rather than on ssDNA. *In vitro*, RecG works as a monomer 69, 70 and efficiently catalyses the re‐pairing of template strands in substrates mimicking replication forks. Interestingly, RecG promoted unwinding reactions occur preferentially on substrates mimicking replication forks with a nascent strand annealed to the lagging‐strand template 9, 15.

RecG catalyses replication fork reversal (also known as replication fork regression) *in vitro* on a substrate containing both nascent strands (Fig. 1A) 9, 10, 11, 12, 13, 14. This RecG‐catalysed replication fork reversal reaction has been observed using an oligonucleotide substrate with nascent strands annealed to both the leading‐ and lagging‐strand templates 14, a replication fork in supercoiled plasmid DNA 71 and a replication fork blocked at a DNA lesion in an *in vitro* replication system where the DNA polymerase and the replicative helicase remain associated with the DNA 11. These studies have led to the opinion that replication fork reversal is an important biochemical activity of RecG 9, 11, 12, 14, 15, 38, 66, 67, 70, 71, 72, 73, 74, 75, 76, 77. RecG can catalyse this reaction thanks to its unusual structure 70. This 76‐kDa enzyme possesses a unique translocation by RecG motif, which is located between the wedge and the helicase domains of the protein and contributes to the unwinding of branched molecules by forming a helical hairpin motif 78. For a more detailed discussion of the structure of the RecG protein, readers are referred to the recent review 38.

## RecG does not catalyse replication fork reversal *in vivo*

**Figure 2:**
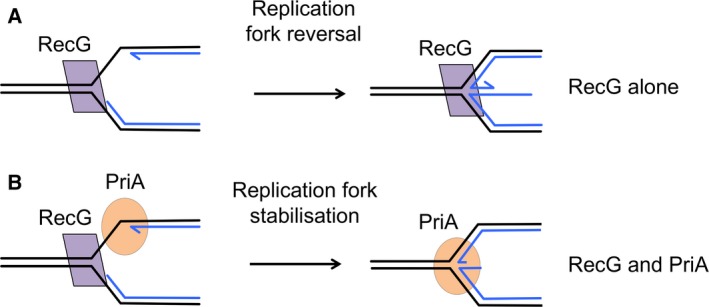
*In vitro* RecG alone catalyses replication fork reversal but RecG and PriA together stabilise the fork. (A) Replication fork reversal *in vitro*. RecG has a preference for replication fork substrates with a 5′ nascent strand at the fork. It binds the double‐stranded template strands and unwinds the new strands by moving the fork backwards. As the template strands re‐pair, the new strands anneal and extrude from the fork, forming a DNA double‐strand end in a replication fork reversal reaction 9, 10, 11, 12, 13, 14, 15. (B) Replication fork stabilisation *in vitro*. When RecG and PriA are both present, RecG begins to re‐pair the template strands while displacing the 5′ ending nascent strand at the fork. PriA is bound to the 3′ ending nascent strand ready to start the reaction to assemble DnaB and initiate DNA replication. The RecG reaction stops when it encounters PriA and the 3′ ending nascent strand. 86.

In 1976, two papers proposed a mechanism for nonmutagenic replication bypass of a DNA lesion that involved reannealing of replicated template DNA strands and extrusion and pairing of newly synthesised DNA strands 55, 57. Over two decades later, a study of *E. coli rep* mutants provided evidence for the occurrence of this replication fork reversal reaction in cells with undamaged DNA but with compromised DNA replication 56. It was proposed that the RecG‐catalysed replication fork reversal reaction observed *in vitro* might also happen following UV irradiation *in vivo* 14. The absence of this pathway in *recG* mutants would permit re‐pairing of template strands to help repair DNA lesions 14. However, none of the studies of replication fork reversal to date, using different ways of compromising DNA replication, has revealed any situation where RecG is required for the reaction *in vivo* 56, 79, 80, 81, 82, 83, 84. Furthermore, a subsequent investigation showed little evidence that RecG promotes replication fork reversal following UV irradiation 85. This generated a conundrum. Why would RecG be so good at catalysing replication fork reversal *in vitro* but unable to catalyse the reaction *in vivo*? A clue to this might be the observation that when PriA is present, RecG initiates the re‐pairing of parental strands but only proceeds as far as bringing the 3′ end of the nascent leading‐strand to the fork junction point, whereupon the DNA is bound by PriA in a fork‐stabilising configuration (Fig. 2) 86. We shall return to this observation later.

## RecG and RuvABC collaborate to stabilise joint molecules during DSBR

As described above, there is good evidence that RecG and RuvABC catalyse alternative steps in the pathway of recombination, which would explain the high DNA damage sensitivity and recombination deficiency of a *ruv recG* double mutant. Since RuvABC is known to act as a branch migration and Holliday junction resolution complex 87, it was attractive to hypothesise that this redundancy arose from two alternative pathways of resolution of Holliday junctions. One possibility was that RecG with the help of a topoisomerase might catalyse the dissolution of structures containing two Holliday junctions as had originally been proposed for bacteriophage lambda recombination 88 and has been shown in eukaryotic chromosomes by a combination of BLM, TopoIIIα and Rmi1 (see 89). However, a substantial proportion of chromosome dimers is generated among recombinants formed in the absence of RuvABC, indicating that crossing over has taken place in conditions where the hypothetical RecG‐mediated resolution pathway would be operating 5, 6. This observation is not compatible with a dissolution pathway catalysed by RecG as topoisomerases do not catalyse crossing over and has prompted two alternative hypotheses. First, an unknown nuclease could participate in the RecG pathway of resolution 5 and second, resolution could be mediated by the next round of chromosomal DNA replication passing through the Holliday junction 6.

**Figure 3:**
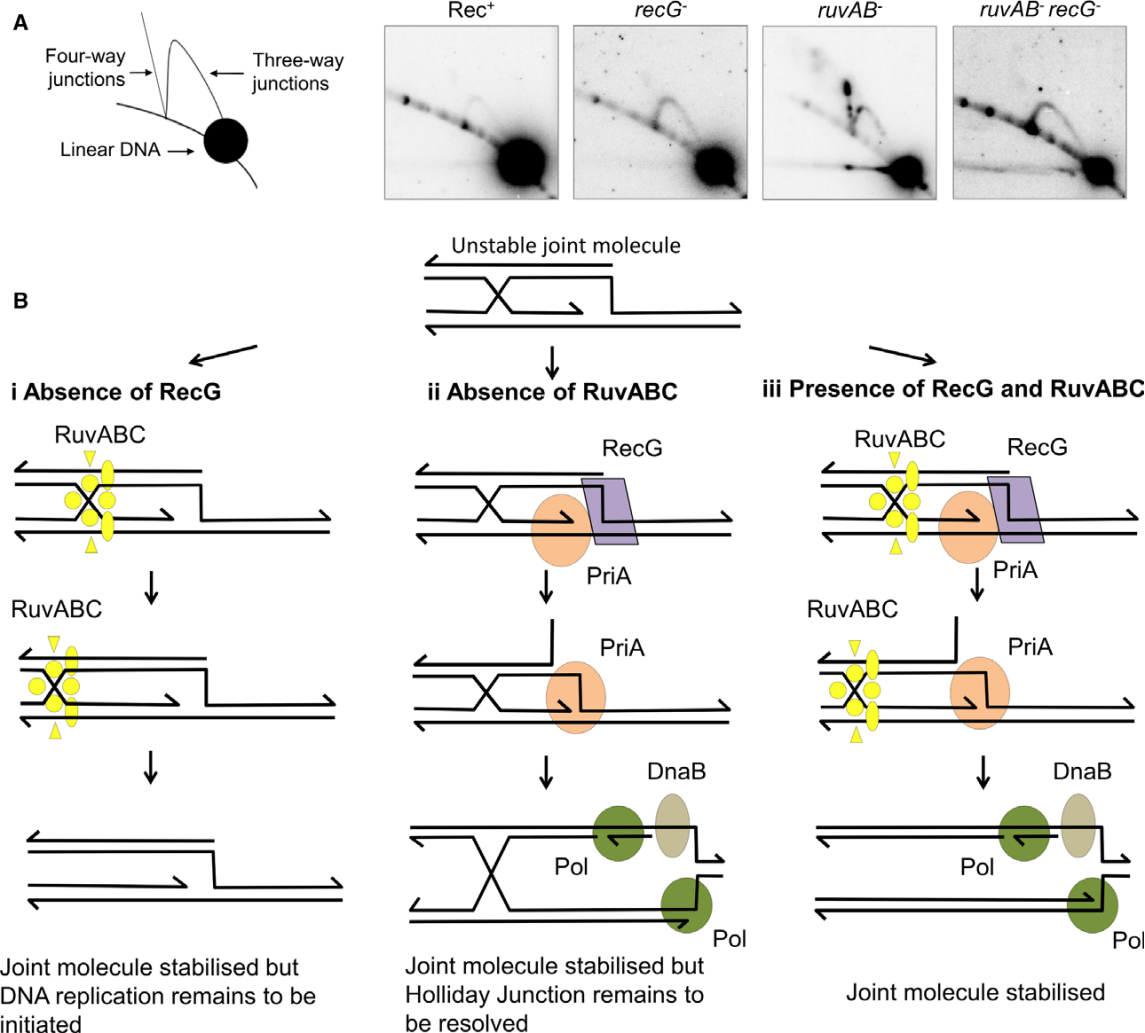
Stabilisation of joint molecules by RuvABC and RecG. (A) DSBR intermediates visualised by 2D gel electrophoresis. RuvAB and RecG do not simply provide alternative pathways for the resolution of Holliday junctions, as previously suggested. Four‐way Holliday junction intermediates accumulate in the absence of RuvAB but not in the absence of RecG. The accumulation of Holliday junctions in the absence of RuvAB requires the presence of RecG 16. Data reproduced with permission from PLoS Genetics. (B) Role of RuvABC and RecG in the stability of joint molecules (i) Joint molecule stabilisation by RuvABC. In the absence of RecG, RuvAB migrates the Holliday junction away from the site of initiation of DSBR and leads to its cleavage by RuvC. Both branch migration and cleavage stabilise the joint molecule. (ii) Joint molecule stabilisation by RecG. In the absence of RuvABC, RecG manipulates the replication fork end of the D‐loop to allow PriA to bind in its 3′ end‐binding fork‐stabilising mode. This allows the initiation of DNA replication that stabilises the joint molecule. (iii) Joint molecule stabilisation by RuvABC and RecG. In the presence of both RuvABC and RecG, both the Holliday junction and replication fork ends of the D‐loop are stabilised.

On the assumption that RuvABC and RecG catalyse alternative pathways of Holliday junction resolution, it was logical to look for evidence of accumulation of Holliday junction intermediates in a *ruvAB recG* double mutant. However, very surprisingly this double mutant failed to accumulate Holliday junction intermediates while a *ruvAB* mutant readily did (Fig. 3A) 16. This result clearly showed that RuvABC is responsible for the resolution of Holliday junctions in cells containing RecG. However, few joint molecules of any kind were detected in a strain lacking both RuvAB and RecG. Clearly, the presence of either RuvAB or RecG is required to generate stable joint molecules (including molecules with Holliday junctions) in the first place 16. This led Mawer and Leach to suggest that the branch migration activities of RuvAB and/or RecG might provide alternative ways of stabilising an initially formed and otherwise unstable form of joint molecule, thus explaining the genetic redundancy observed previously. Since joint molecules could not be stably recovered in the absence of RuvAB and RecG, it was hypothesised that initially formed intermediates generated in the absence of these proteins might consist of D‐loops that could be destabilised by a helicase. Further work revealed that this helicase is PriA 17.

The stabilisation of initially formed joint molecules, consisting of D‐loops generated by the RecA protein, through the branch migration activities of RuvAB and RecG is readily understandable. Given that RuvAB branch migrates Holliday junctions prior to their resolution by RuvC, it is highly probable that the stabilising activity of RuvABC operates at the Holliday junction end of a D‐loop by extending the region of base pairing between the recombining duplexes, leading to their covalent exchange following cleavage and ligation (Fig. 3Bi). However, the site of action of RecG is less clearly defined by the biochemistry of the enzyme, since this protein can catalyse both the migration of Holliday junctions and the remodelling of replication forks. During DSBR both of these structures are present, one at each end of a D‐loop. A clue as to the nature of the RecG substrate *in vivo* comes from the observation that a class of suppressors of the *recG* recombination‐deficient phenotype carries mutations in PriA, either reducing or eliminating the helicase activity of the protein 90. PriA plays a critical role in the reloading of DnaB, the replicative helicase, onto various DNA structures 91, 92, 93, 94. It does so by binding to a replication fork substrate with a 3′ end at the fork junction in a configuration whereupon the fork is stabilised and the helicase activity of PriA is switched off 95. The helicase‐defective mutants of *priA* that suppress the recombination‐deficient phenotype of *recG* mutants are indeed competent for catalysing replication restart 96. This suppression, coupled with the observation that RecG delivers PriA to a replication fork substrate in its 3′ end‐binding mode 86, argue strongly for a joint molecule stabilising role of RecG associated with the replication fork end of a D‐loop (Fig. 3Bii). Accordingly, we propose that D‐loops are stabilised in the presence of RuvABC and RecG by activities at both DNA junctions (Fig. 3Biii). Furthermore, we conclude that this overlap in function could be responsible for the genetic redundancy of *recG* and *ruvABC* mutants.

## RecG controls DNA amplification during DSBR and at arrested replication forks

It has long been known that there is a link between RecG and DNA replication. cSDR is induced in the absence of RecG 97. cSDR is a form of DNA synthesis 98, 99 that requires RecA 100, 101, transcription 102, 103 and is stimulated in *rnhA* mutants 102. It is therefore proposed to originate from persistent R‐loops that may be generated through the action of RecA. *recG rnhA* double mutants are not viable and it has been proposed that RecG either unwinds persistent R‐loops or prevents their formation through opposing the action of RecA 97. Inducible stable DNA replication (iSDR) is also elevated in the absence of RecG 104, 105. iSDR requires the induction of the SOS response 106, the action of RecBCD 105, 107 and is insensitive to inhibition of transcription 108, consistent with resulting from DSBs. The reader is directed to the review 108 for a more detailed description of cSDR and iSDR.

During DSBR in *E. coli,* the RecBCD enzyme resects broken ends for distances of up to several kilobases 109. It is therefore essential that the degraded DNA is restored. This is normally carried out by establishing DNA replication initiated through the action of PriA 60, arguing for the loading of the replicative helicase DnaB and the replicative DNA polymerase PolIII. However, in the absence of RecG, DNA over‐replication is observed following DNA damage 19, 20, 21. At a site‐specific DNA break, this over‐replication flanks the site of DSBR 17. Furthermore, even in the absence of DNA damage, *recG* mutants over‐replicate the terminus region of their chromosome between termination sites *terA* and *terB* 17, 18, 20, 22, 23. This over‐replication is mediated by PriA and PriB and is suppressed by combining the *recG* mutation with PriA‐helicase mutations 22. These results suggest that the replicative helicase DnaB loads onto DNA substrates generated in this region.

Four alternative hypotheses have been proposed, none of which is free from limitations, to explain the observation that DNA amplification is prevented by RecG.

**Figure 4:**
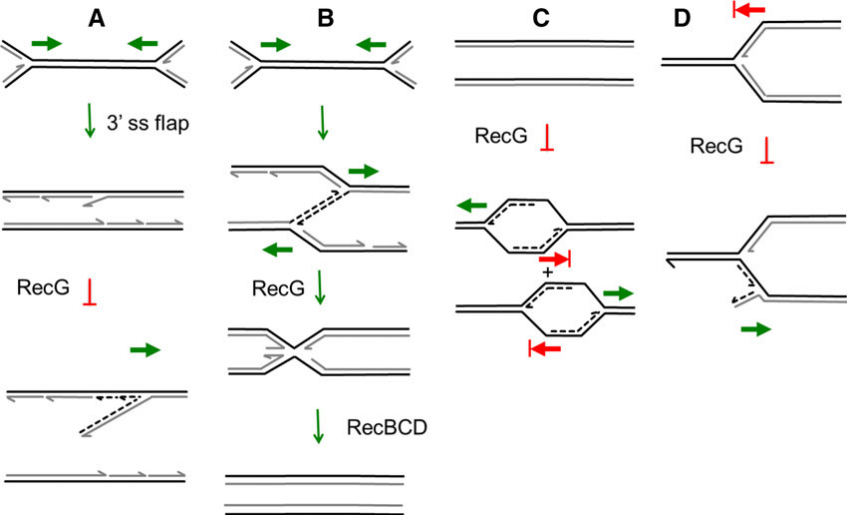
Four different models proposed to explain how RecG controls DNA amplification. (A) Fork collision and restart at a 3′ flap. When two replication forks (moving in the directions of the green arrows) collide, it is hypothesised that in the absence of RecG a 3′ flap is generated that leads to the assembly of a replication fork. In the presence of RecG, the 3′ flap is converted into a 5′ flap that can be degraded by 5′–3′ exonucleases 18, 19, 21, 22. (B) Fork collision and template‐switching followed by replication fork reversal. When two replication forks (moving in the directions of the green arrows) collide, it is hypothesised that template switching occurs leading to over‐replication. This is corrected by RecG‐dependent replication fork reversal and DNA degradation at one (or both) of the replication forks 23. (C) cSDR and termination at Tus/*ter* blocks. It is proposed that, in the absence of RecG, cSDR initiates at sites of transcription around the genome leading to replication forks that are blocked by Tus/*ter*. This results principally in over‐replication of the region between termination sites (at the positions of blocked red arrows) as cSDR forks are removed by colliding with origin‐initiated replication forks 25. (D) Reverse‐restart of an arrested replication fork. At an arrested replication fork (at the position of the blocked red arrow) RecG prevents the assembly of the replicative helicase on the newly synthesised lagging‐strand. In the absence of RecG, this loading is permitted and backwards‐directed DNA replication occurs 17.

First (Fig. 4A), DNA amplification is a consequence of DNA flaps that are hypothesised to arise when replication forks collide 18, 19, 21, 22. It is hypothesised that replication fork collisions frequently give rise to 3′ flaps that can be converted into 5′ flaps by RecG, and then these 5′ flaps are degraded by 5′–3′ exonucleases 18, 21. In the absence of RecG, the 3′ flaps persist and are converted into new replication forks through the action of PriA 18, 21. The existence of 3′ flaps is supported by the preference of RecG for processing 3′ flaps over 5′ flaps 15, 86 and the observation of DNA over‐replication in the terminus region of the chromosome of a triple 3′–5′ exonuclease mutant, *xseA xonA sbcDC* 22. The products of the *xseA*,* xonA* and *sbcDC* genes (exonuclease VII, exonuclease I and SbcCD exo/endonuclease respectively) are the major 3′–5′ exonucleases in *E. coli*. They participate in several DNA repair and genome stability pathways and the reader is directed to review 110 for a more detailed discussion of their functions. The over‐replication in the *xseA xonA sbcDC* mutant is very interesting and does indeed suggest the existence of a pathway of DNA amplification involving 3′ overhangs. However, contrary to the prediction of the model that RecG can remove 3′ flaps by converting them to 5′ flaps, RecG is unable to prevent this pathway as the amplification observed in the *xseA xonA sbcDC* mutant occurs in the presence of RecG. Conversely in the absence of RecG, the three 3′–5′ exonucleases are not able to prevent over‐replication. Therefore, either a single 3′ flap processing pathway is delicately balanced between the activities of RecG on one hand and the three 3′–5′ exonucleases on the other, or there are two separate pathways operating on different substrates. The synthetic lethality of a *recG xseA xonA sbcDC* quadruple mutant provides some indirect evidence for the existence of a single substrate but it is not conclusive since the phenotype of DNA over‐replication in the terminus region is not lethal and the cause of lethality of the quadruple mutant is unknown 18. Furthermore, although a *priA300* helicase defective mutation suppresses the DNA damage sensitivity of a *recG* mutant, it does not suppress the DNA damage sensitivity of an *xseA xonA sbcDC* mutant 18, presenting a counter‐argument in favour of the existence of two distinct substrates. In this first model, PriA is hypothesised to recruit DnaB without acting in PriA's 3′ end‐binding and fork‐stabilising mode, which does not fit easily with the biochemical observation that RecG remodels a replication fork substrate to favour PriA binding in its 3′ end‐binding mode 86. The DNA ends generated during this process should be at multiple positions where collisions happen between replication forks and should be pointing in both directions but in fact they are primarily generated at *ter* sites where they are unidirectional 17. Finally, a complete inversion of chromosome replication is observed in a dnaA recG tus rpo* mutant 22 where replication forks cannot form at the origin of DNA replication so there is no prediction of fork collisions in the chromosome terminus region, from where replication is nevertheless observed to originate.

Second (Fig. 4B), DNA amplification is caused by replication forks sliding past each other in the terminus region of the chromosome 23. This reaction is corrected by RecG that catalyses replication fork reversal on one (or both) of the replication forks, generating one or more DNA double‐strand ends that can be degraded by RecBCD. This hypothesis differs from the first hypothesis in two principal respects. First, RecG is predicted to generate DNA double‐strand ends rather than to remove a precursor of DNA double‐strand ends and second the sliding of replication forks past each other requires a rather complex double DNA template switch. We now know that there is an increase in the frequency of DNA double‐strand ends that bind RecA protein in the terminus region of the chromosome of a *recG* mutant 17, which is not predicted by this model. As with the first hypothesis, this model does not explain the inversion of chromosome replication observed in a dnaA recG tus rpo* mutant, since this model also predicts that over‐replication of the terminus region requires the meeting of replication forks coming from the origin, which are absent in this mutant 22.

Third (Fig. 4C), DNA amplification in the terminus region is simply a consequence of cSDR that is allowed to occur in a *recG* mutant and proceeds through the terminus region until it reaches a Tus/*ter* block 25. cSDR may indeed contribute in some ways to the pattern of DNA replication observed in a *recG* mutant. However, this hypothesis does not explain the origin of the DNA double‐strand ends that bind RecA at *ter* sites in a *recG* mutant 17. Furthermore, the unusual replication observed in a *recG* mutant is different from that observed in an *rnhA* mutant as only the former can be suppressed by a *priA300* helicase‐defective mutant 111. These observations argue against the involvement of cSDR in the terminus over‐replication formed in the absence of *recG*. In contrast, the stimulation of iSDR in a *recG* mutant could be related to the over‐replication observed in the absence of RecG as proposed by the first and fourth hypotheses. iSDR occurs as a consequence of DSBR by homologous recombination and the recombination deficiency of *recG* mutants is known to be suppressed by *priA300* 112.

Fourth (Fig. 4D), DNA amplification is caused by the incorrect loading of PriA at a site of replication fork arrest or at a newly formed replication fork 17, leading to the formation of a backwards‐directed replication fork. This reverse‐restart hypothesis is based on two observations. (a) RecG loads PriA onto a model replication fork in the 3′ end‐binding and fork‐stabilising mode 86, predicted to facilitate the loading of DnaB to restart the fork correctly. (b) DNA double‐strand ends bound to RecA protein are detected at the sites of initiation of DNA amplification at an induced DSB and in the terminus region of the chromosome between *terA* and *terB* 17. As attractive as this model is, it does not explain all the previous observations either. For example, it does not explain the observation of DNA amplification in the terminus region of a RecG^+^ cell in the absence of the 3′–5′ exonucleases. It also does not directly explain the inversion of chromosome replication observed in a dnaA recG tus rpo* mutant 22. However, DSBs have been observed surrounding the *dif* site 17, 113. These breaks could provide the DNA replication initiation sites that would allow this inversion of chromosome replication to occur according to this model.

Only the first and fourth hypotheses propose that over‐replication occurs as a consequence of DNA double‐strand ends that are generated in the absence of RecG. The detection of RecA bound to DNA double‐strand ends in the terminus region, which is specifically enhanced in a *recG* mutant, provides support for these two models. This stimulation of DSBR is also consistent with iSDR being induced in a *recG* mutant.

## Conclusions and perspectives

It is clear that RecG prevents DNA amplification at a site of induced DSBR in the *lacZ* gene 17. This is also the case in the terminus region of the *E. coli* chromosome where DNA amplification in the absence of RecG is similarly associated with DSBR 17. These observations are only in accordance with hypotheses one and four (Fig. 4A,D). We favour the simple explanation, prevention of reverse‐restart, that is described in Fig. 4D. RecG directs the correct loading of PriA, at replication forks that have lost (or not yet acquired) the DNA replication machinery. Appropriate binding of PriA allows DNA replication to proceed correctly via loading of the replicative helicase DnaB. In the presence of RecG, the formation of normal replication forks is predicted to occur at sites of DSBR where they are required to replace the DNA lost during resection. In the absence of RecG, PriA and DnaB can be loaded incorrectly to replications forks that have been created by DSBR or replication forks that have arrested and lost their replisomes. Incorrect loading of DnaB leads to DNA amplification (Fig. 4D) 17.

However, if this explanation is not correct and DNA double‐strand ends arise as a consequence of replication fork collisions in the absence of RecG (Fig. 4A), then these collisions must occur primarily at *terA* and *terB* sites in a *recG* mutant as this is where RecA binding to DNA double‐strand ends is detected by ChIP 17. The ChIP data reveal that RecA binding is at one‐ended DNA breaks all pointing in one of the two possible directions at each of the *ter* sites 17. This implies that any fork collision occurring at a *ter* site would have to lead to a specific orientation of break. This may be possible if the direction of replication fork movement upon collision with a *ter* site can determine the strand on which the hypothetical 3′ single‐strand is generated.

Why a *xseA xonA sbcDC* triple 3′–5′ exonuclease mutant stimulates DNA amplification in the terminus region of the chromosome remains to be determined. Does this amplification arise from the same pathway as the over‐replication in a *recG* mutant, or is it mediated by a separate pathway controlled by 3′ overhangs? How DNA replication is initiated in the terminus region of a dnaA recG tus rpo* mutant also remains to be determined. Is this replication initiated by the DSBs detected on the two sides of the *dif* site 17, 113? Further investigations are required to answer these questions.

DNA replication restart is stringently restricted in eukaryotic cells. However, one might predict that such a pathway could exist to ensure completion of replication between the most telomere proximal origin of replication and the end of the chromosome. One might also predict that, even in the absence of a pathway for restart, incorrect loading of a replicative helicase at the site of a stalled replication fork, to allow reverse‐restart, should be prevented to avoid DNA amplification. Perhaps this is where SMARCAL1 plays a role in maintaining genome stability.

## Author contributions

Both authors have contributed to writing the manuscript.
